# The rice cellulose synthase-like D4 gene (*OsCSLD4*) is required for resistance to *Xanthomonas* pv. *oryzae*

**DOI:** 10.7717/peerj.21562

**Published:** 2026-09-09

**Authors:** Lu Shi, Guotian Li, Mawsheng Chern, Rashmi Jain, Pamela C. Ronald

**Affiliations:** 1 Department of Plant Pathology and Genome Center, University of California, Davis, CA, United States of America; 2 Feedstocks Division, Joint BioEnergy Institute, Lawrence Berkeley National Laboratory, Berkeley, CA, United States of America; 3 Jiangsu Key Laboratory for Food Quality and Safety-State Key Laboratory Cultivation Base, Ministry of Science and Technology, Jiangsu Academy of Agricultural Science, Nanjing, Jiangsu, China; 4 National Key Laboratory of Agricultural Microbiology and College of Plant Science and Technology, Huazhong Agricultural University, Wuhan, Hubei, China

**Keywords:** Rice, *XA21*, Cellulose, Reactive oxygen species, *Xanthomonas oryzae* pv. *oryzae*, Plant defense

## Abstract

Plant cell walls serve as a physical support and a barrier to pathogen invasion. Cellulose is the main component of cell walls. The *cellulose synthase-like D* (*CSLD*) subfamily genes are required for plant normal development. In rice, *Oscsld4* mutant plants are dwarfed and have narrow, rolled leaves. The role of *OsCSLD4* in rice immune responses is unclear. We carried out a forward genetic screen using rice mutants expressing the XA21 immune receptor to identify components required for the resistance to *Xanthomonas oryzae* pv. *oryzae* (*Xoo*). One mutant from the screen carries a loss-of-function mutation in *OsCSLD4*. *OsCSLD4* is required for resistance to *Xoo* mediated by both the XA21 and XA26 immune receptors and also participates in the basal resistance to *Xoo*. Hallmarks of the XA21-mediated immune response, including induction of the defense marker gene *KO5*, reactive oxygen species (ROS) burst and the phosphorylation of mitogen-activated protein kinases (MAPKs), are not compromised in the *Oscsld4* mutant. These findings suggest that OsCSLD4 does not function as a core signaling component of the immune receptor pathway, but rather maintains the structural integrity of the cell wall as an effective physical barrier. This structural defense is essential for the full manifestation of both innate and receptor-mediated immunity.

## Introduction

Plants have evolved structural and physiological defense mechanisms against pathogenic microbes. The plant cell wall serves as a physical barrier that impedes the invasion of pathogens and reduces their contact with the plant cells (Wolf, 2022). In addition, plants are able to produce a variety of factors with anti-microbial activity, such as reactive oxygen species (ROS) and pathogenesis-related proteins (Jones, Staskawicz & Dangl, 2024). Both the structural and physiological aspects of defense can be enhanced upon pathogen perception by immune receptors, which activate defense signaling pathways (Chisholm et al., 2006). Cell wall strengthening is typically achieved by deposition of lignin and/or callose (Zhong & Ye, 2007).

The main load-bearing polymer of plant cell walls is cellulose. Cellulose is a paracrystalline structure assembled from individual *β*-1,4-glucan chains that are synthesized by mobile cellulose synthase (CESA) at the plasma membrane from glucose (McFarlane, Döring & Persson, 2014). The individual *β*-1,4-glucan chain can crystallize into cellulose microfibrils, which form rosette terminal complexes. The rosette terminal complexes are hexameric protein structures, approximately 25 nm in diameter (Guerriero, Fugelstad & Bulone, 2010). Cellulose deficient mutant plants often show increased sensitivity to abiotic stress (Wang, McFarlane & Persson, 2016; Zhao et al., 2022). For example, loss of *AtCESA8* led to enhanced drought and osmotic stress tolerance in *Arabidopsis* (Chern et al., 2005). While the functions of cellulose in plant development during abiotic stresses have been determined, their role in rice immunity remains relatively unexplored. Consequently, mutations affecting cell wall biosynthesis may not only cause developmental defects but also potentially compromise the plant’s overall ability to restrict pathogen movement, regardless of the activation of specific immune pathways.

The rice *Xa**nthomonas resistance 21* (*XA21*) gene was first identified due to its ability to confer robust resistance to a broad-spectrum of strains of the rice bacterial leaf blight pathogen *Xanthomonas* pv. *oryzae* (*Xoo*) (Song et al., 1995). The *XA21* gene, encoding a plasma membrane-localized receptor kinase containing an extracellular leucine-rich repeats domain, a transmembrane region, and an intracellular kinase domain, is representative of a large family of immune receptors structurally conserved among plants and animals (Couto & Zipfel, 2016). XA21 is activated upon recognition of the *Xoo-* derived, sulfated microbial peptide, named RaxX (required for activation of XA21-mediated immunity X) (Pruitt et al., 2015).

To identify factors that are essential for the effective restriction of *Xoo* in rice, we utilized the XA21-mediated immunity system as a sensitive background for a forward genetic screen. While such screens are designed to find components of receptor-mediated signaling, they also frequently uncover broader physiological or structural factors required for the final expression of resistance. Here, we report that the rice *cellulose synthase-like D4* (*OsCSLD4*) gene, identified through this screen, is a key determinant of rice resistance. Our results indicate that the increased susceptibility of *Oscsld4* mutants to *Xoo* is likely due to a compromised cell wall barrier rather than a defect in the core immune signaling machinery.

## Materials & Methods

### Plant material and growth conditions

Kitaake, a photoperiod-insensitive cultivar of *japonica* rice (*Oryza sativa* ssp. *japonica*) and KitaateX, a Kitaake variety carrying the *XA21* gene with maize ubiquitin promoter (Song et al., 1995; Li et al., 2017; Jain et al., 2019). Rice mutant FN2572 was selected by screening a mutant population induced by fast-neutron (FN) irradiation in the KitaakeX background with *Xoo* (Li et al., 2017). The W481 mutant was initially identified based on the distinct leaf and whole plant morphology shared with FN2572.

Rice plants were grown and maintained in the greenhouse facility at the University of California, Davis, and transported into controlled growth chambers at the 5–6-week-old stage for *Xoo* inoculation. The controlled growth chambers were set to ∼28 °C, 85% humidity and 14/10 day/night regime.

### *Xoo* inoculation, leaf lesion measurements and *in planta* bacterial growth curve analysis

*Xoo* strains PXO99 and PXO79 were grown on PSA plates in dark for 3 days at 28 °C. The bacteria were resuspended in sterile water and adjusted to an OD_600_ of ∼0.5 (5 × 10^8^ CFU/mL). Rice plants were inoculated by using the scissor clipping method (Sha et al., 2023). Disease lesions were scored at 14 days after *Xoo* inoculation.

For *in planta* bacterial growth analysis, plants were inoculated as described above. The protocol for the measurement of bacterial growth curve was as previously reported (Chern et al., 2005; Luu et al., 2019). Rice leaves were cut into sections about 10-cm from the site of inoculation at four timepoints (0, 4, 8, 12 dpi). Two leaves from each tiller, representing one biological replicate, were cut into two mm pieces and incubated in sterile water at 28 °C with shaking at 230 rpm for 2 h. This suspension was then serially diluted to 10^−4^, plated on PSA plates with cephalexin (20 μg/mL), and incubated at 28 °C. Two days later the colonies were counted and the colony forming units per leaf was calculated. Three biological replicates, each representing the mean of four technical replicates, were measured for each data point.

### Cloning and complementation

The complete *OsCSLD4* (LOC_Os12g36890) gene (6,163 bp), including the full-length coding sequence, its promoter (1,776 bp), and 3′ untranslated/untranscribed sequence (487 bp), was amplified and cloned into the pENTR/D TOPO vector. After the sequence is confirmed by sequencing, the gene was introduced into the Gateway-compatible vector pCAMBIA2300. Then the construct was transferred into the *Agrobacterium tumefaciens* strain EHA105 by electroporation.

For functional complementation, the pCAMBIA2300 binary vector carrying the *OsCSLD4* gene was introduced into rice calli induced from the mature embryos of mutant line FN2572 by *Agrobacterium (EHA105)* infection. Rice transformation was carried out according to a protocol described before (Cheng, Sardana & Altosaar, 1998) with some modifications. Briefly, Agrobacterial colonies were inoculated in two mL of LB medium containing kanamycin (50 mg/L) and grown overnight; an aliquot of the overnight culture was used to inoculate ∼10 mL of TY medium containing 200 μM acetosyringone plus 50 mg/L kanamycin and the culture allowed to grow 4–6 h until OD_600_ reached 0.1–0.2. Calli were incubated with the Agrobacterium culture for 30 min at room temperature with gentle shaking. Calli were blotted on sterile Whatman paper four times until dry to remove excess bacteria. Then, the calli were transferred to MS (Murashige and Skoog) agar (1.5%) medium with 2,4-dichlorophenoxyacetic acid (2,4-D) at 2 mg/L, sorbitol (50 g/L) and acetosyringone (200 μM) to cocultivate for 3 days in the dark at 22 °C. For selection, calli were moved to MS agar (0.8%) medium with 2,4-D (2 mg/L), carbenicillin (400 mg/L), timentin (200 mg/L), PPM (plant preservative mixture, 1,000X dilution of stock), and G418 (100 mg/L). For regeneration, calli were moved to MS agar (1.5%) medium with sorbitol (50 g/L), 6-benzylaminopurine (1.8 mg/L), 1-naphthaleneacetic acid (0.3 mg/L), PPM, carbenicillin (400 mg/L), timentin (200 mg/L), and G418 (100 mg/L). After approximately one month in selection and one month in regeneration media, regenerated plantlets were transferred to rooting medium (MS medium with G418). Plants were transferred to soil when roots were well developed (in about 10 days).

### RNA extraction and RT-qPCR

Total RNA was extracted from rice leaf tissues using the TRIzol reagent (Invitrogen, Waltham, MA, USA). Then the total RNA was treated with the TURBO DNA-free kit (Ambion, New York, NY, USA) to remove residual genomic DNA and stored in −80 °C. The concentration was measured with a NanoDrop spectrophotometer (Thermo, Waltham, MA, USA). For each RNA sample, two µg of total RNA were used for cDNA synthesis using the High-Capacity cDNA Reverse Transcription Kit (Applied Biosystems). Reverse transcription quantitative PCRs (RT-qPCRs) were performed on a Bio-Rad CFX96 Real-Time System (Bio-Rad, Hercules, CA, USA) using 2X SYBR Green I Master Mix in a total volume of 20 µL per reaction. The 2^(−ΔΔ*Ct*)^ method was used to calculate the relative changes in gene expression normalized to the rice *actin* gene expression level. RT-qPCR reactions were run for 40 cycles with annealing and amplification at 58 °C for 5 s and denaturation at 95 °C for 5 s. The primers see Table S1.

### ROS assays and treatment of rice leaf clippings with RaxX21-sY peptide

Analysis of the ROS burst in rice was performed as previously described (Thomas et al., 2018) with minor modifications. We used 4- to 5-week-old rice plants (Kitaake, KitaakeX, FN2572 and W481) grown in the greenhouse. Well-developed rice leaves were cut into small pieces (1 ×3 mm) and floated on autoclaved water in circular petri dish overnight at clean hood with light to remove the wounding response. Then leaves were transferred to 96-well plate containing 100 μL elicitation solution (80 μL ddH_2_O +10 μL 10 ×Lumi-Master mix). The 10 × Lumi-Master mix contain 200 μM L-012 and 0.01 mg/mL HRP (Horseradish Peroxidase) solution. Lastly, 10 μL RaxX21-sY or dd H_2_O was added into each well using a multi-channel pipet right before the start of ROS assay program (Luminometer). ROS production was continuously measured for 1 s per reading with a high sensitivity plate reader (TriStar, Berthold, Germany); the total process takes 3 h.

### Peptide treatment, protein extraction, and western blot assays

RaxX-sY peptide treatments of detached rice leaves were performed using 5-week-old rice. The leaf clippings (1.5–2 cm) prepared from expanded adult leaves were placed into a 6-well cell culture plate, and kept under constant light overnight; samples were treated with 500 nM RaxX-sY or ddH_2_O in the following day (Thomas et al., 2016).

Leaf tissues were harvested after treatment for 30 min with RaxX-sY, and frozen in liquid nitrogen for total protein extraction, according a protocol previously described (Liu et al., 2020). Protein was separated on an 10% sodium dodecyl sulfate-polyacrylamide gel and blotted onto a PVDF membrane (Biorad, Hercules, CA, USA). On Western blot, the XA21 was detected by using a Myc antibody (Invitrogen, Waltham, MA, USA) following the method described previously. Phosphorylated MAP kinases were detected by probing with anti-p42/44 MAPK primary antibodies (1:1000, Cell Signaling Technology) overnight, followed by rabbit-HRP conjugated secondary antibodies (Sigma) (Schwessinger et al., 2015).

### Statistical analysis

All statistical analyses were conducted with GraphPad Prism 10 (GraphPad Software), with the exact methods and parameters indicated in each figure.

## Results

### Two morphologically similar rice mutants exhibit defects in *XA21*-mediated immunity

**Figure 1:**
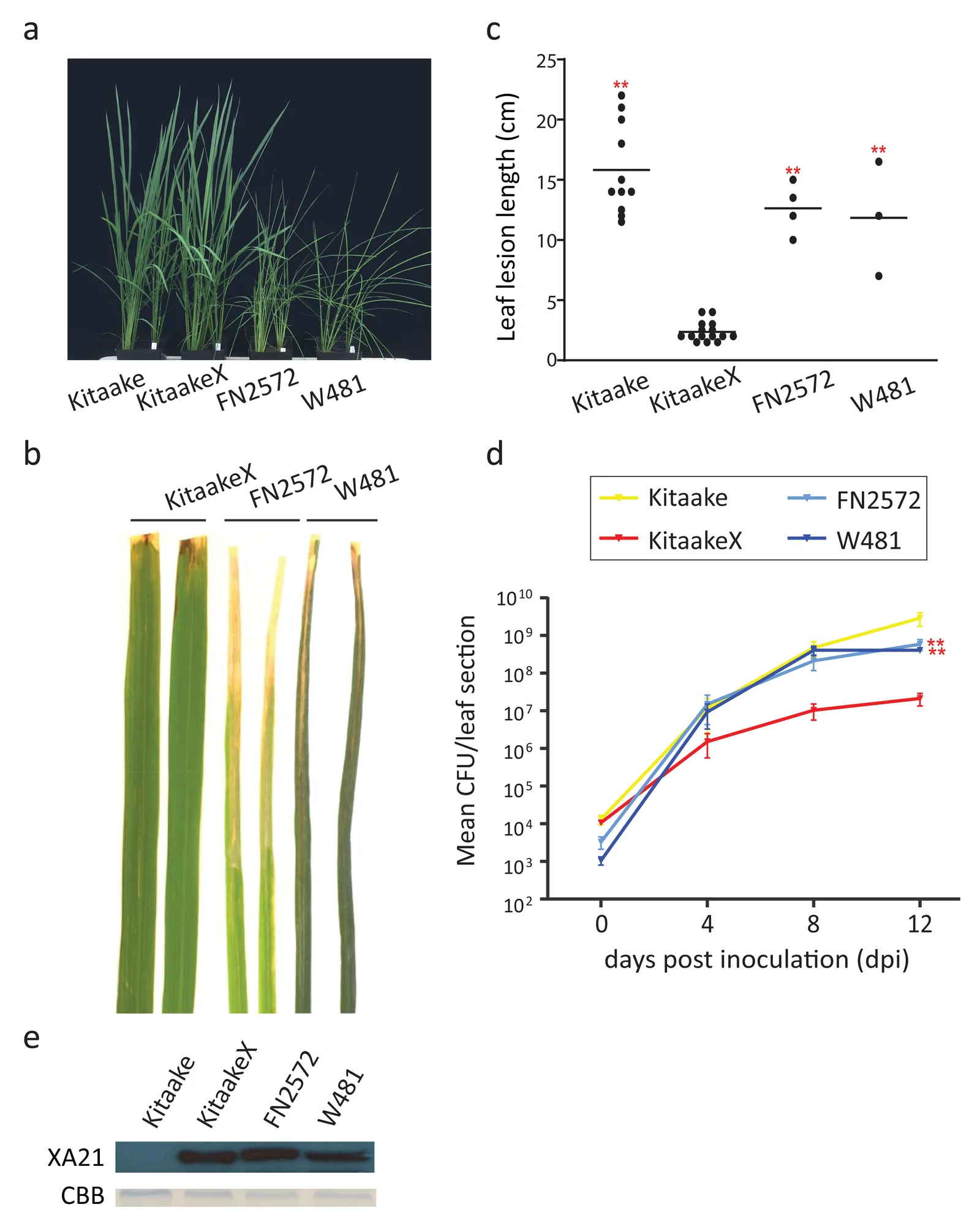
The *Xa21*-mediated resistance to *Xoo* is compromised in rice mutants FN2572 and W481. (A) The morphology of five-week-old Kitaake, KitaakeX, FN2572, and W481. (B) Photograph of rice leaves fourteen days after *Xoo* inoculation. (C) Lesion lengths of the leaves fourteen days after *Xoo* inoculation. Data points represent lesion lengths for 3–15 leaves. (D) Bacterial growth curves after *Xoo* inoculation. Bacterial populations were counted 0, 4, 8, 12 days post inoculation (dpi). Error bars represent standard deviations (*n* = 4). Red asterisks in (C) and (D) indicate significant differences from KitaakeX plants using Dunnett’s test (*P* < 0.01). Kitaake and KitaakeX were included as the susceptible and the resistant control, respectively. (e) Protein extracts were probed using a Myc (for XA21) antibody in western blot analysis. Coomassie Brilliant Blue (CBB) staining of the membrane was included as a loading reference.

To identify the genes required for *XA21*-mediated immunity, we screened 21,000 fast-neutron-irradiated KitaakeX M_2_ plants, representing approximately 3,000 independent M_1_ lines, for suppressors of *XA21*-mediated immunity (*sxi*) to *Xoo* (Chen et al., 2021; Liu et al., 2022; Sha et al., 2023). Here, we report that the *sxi6* mutant (line FN2572) displays susceptibility to *Xoo* compared with the parental line, KitaakeX, which expresses the *Xa21* gene under control of a maize ubiquitin promoter in the Kitaake background (Jain et al., 2019). The *sxi6* mutant displays narrow, rolled leaves and dwarfism compared to the KitaakeX parent and Kitaake plants, which do not contain the *Xa21* gene (Fig. 1A). We subsequently identified another mutant line (line W481) that showed distinct leaf and whole plant morphology similar to FN2572, including narrow, rolled leaves and dwarfism, and thus included it in our characterization of *sxi6*.

In response to *Xoo* inoculation, the leaves of both the FN2572 and W481 plants developed long water-soaked lesions (approximately 12 cm) typical of the disease two weeks post inoculation, whereas KitaakeX rice plants showed short lesions, approximately two cm long (Figs. 1B, 1C). Although the *oscsld4* mutants exhibit a dwarf phenotype with shorter leaves, these ∼12 cm lesions in FN2572 and W481 cover the majority of the leaf blade, indicating a near-complete loss of the restriction normally imposed by XA21. Moreover, bacterial growth curve analysis revealed that the *Xoo* bacterial populations in mutant FN2572 (6 × 10^8^ CFU/leaf) and W481 (4 × 10^8^ CFU/leaf) plants are approximately twenty to thirty-fold greater than in the KitaakeX parent (2 × 10^7^ CFU /leaf) at 12 days post inoculation with *Xoo* (Fig. 1D). These results demonstrate that the *XA21*-mediated immunity is impaired in the FN2572 and W481 mutant lines. To test whether impaired plant immunity in line FN2572 and W481 is caused by alteration of XA21 expression, we conducted western blotting assays and found no change in the XA21 protein level (Fig. 1E).

### The loss of *Xa21*-mediated immunity in rice mutants FN2572 and W481 is caused by loss-of -function mutations in *OsCSLD4*

To identify mutations in FN2572 and W481, we carried out whole genome sequencing (WGS) of both FN2572 and W481 (see FN2572-S and W481-5-1 in the Kitbase database, https://kitbase.ucdavis.edu/). Mutant W481 contains three large deletions covering seven genes based on the MSU rice genome annotation version 7 (http://rice.plantbiology.msu.edu/cgibin/gbrowse/rice/). To assess which deletion is correlated with the susceptible phenotype, we backcrossed the W481 mutant with its parental line KitaakeX to generate a segregating progeny population. By analyzing their phenotypes and genotypes, we found that the susceptibility of mutant W481 co-segregates with a 30 kb deletion on chromosome 12 (Fig. S1). This deletion truncates four genes encoding a cellulose synthase-like D4 (OsCSLD4, LOC_Os12g36890) protein, a putative drought-induced 19 protein (LOC_Os12g36900), and two putative calmodulin binding proteins (LOC_Os12g36910 and LOC_Os12g36920) (Fig. S2A). Whole genome sequencing data indicate that mutant FN2572 contains a 4 bp deletion (CTGG) in the same *OsCSLD4* (LOC_Os12g36890) gene that is affected in W481 (Fig. S2B). Based on these results, we hypothesized that the *OsCSLD4* gene is responsible for the morphological changes and impairment of *Xa21*-mediated immunity observed in the two mutants.

**Figure 2:**
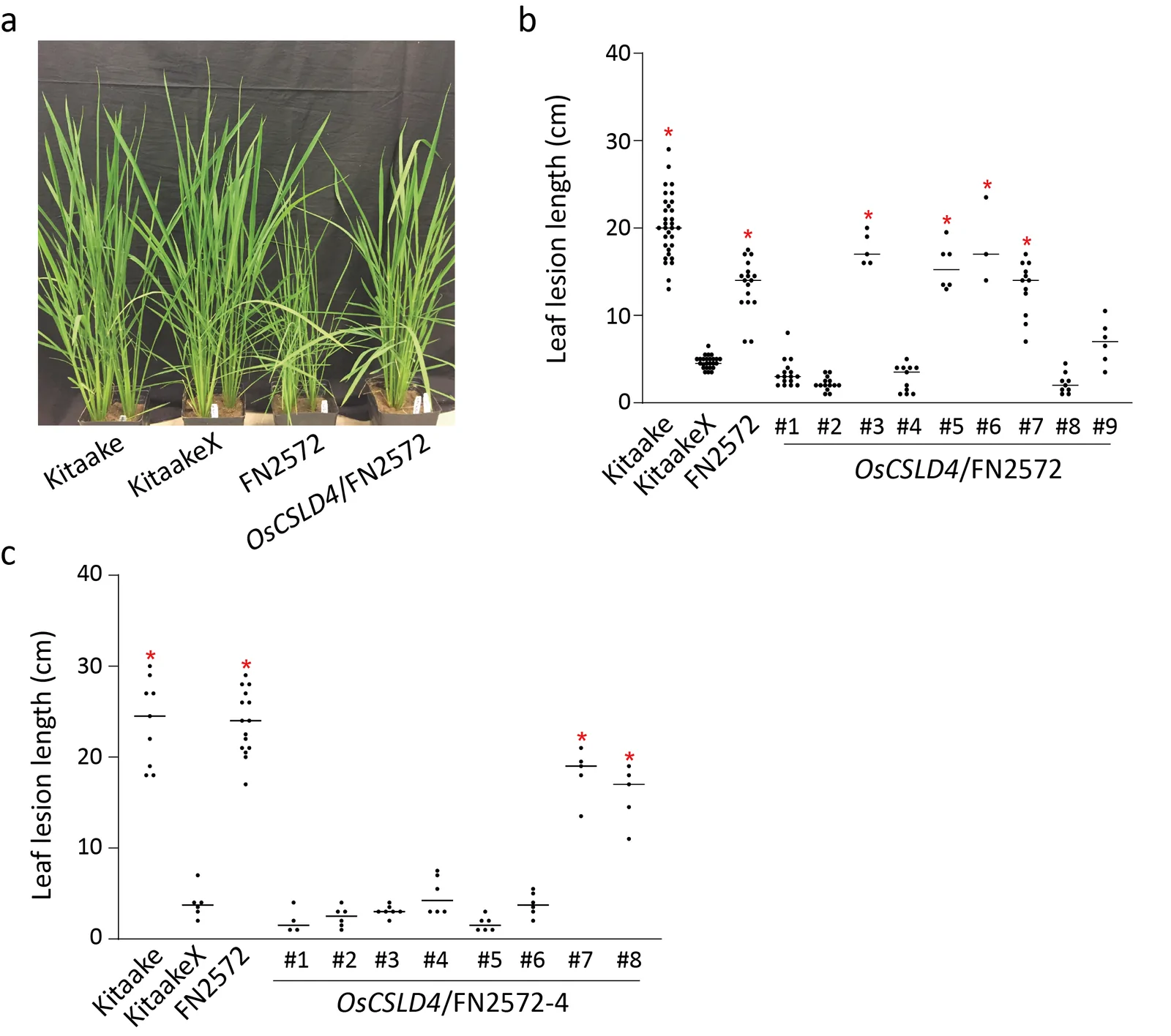
*OsCSLD4* complements the FN2572 susceptible phenotypes. (A) The morphology of five-week-old Kitaake, KitaakeX, FN2572, and FN2572 plants transgenically expressing *OsCSLD4*. (B) Lesion lengths of *Xoo*-inoculated leaves from Kitaake, KitaakeX, FN2572, and nine independent T0 complementation lines (*OsCSLD4*/FN2572). Leaves were measured 14 days after inoculation with *Xoo*. (C) Leaf lesion lengths of eight T1 siblings derived from *OsCSLD4*/FN2572 T0 line #4 (*n* = 3–32). Red asterisks in (B) and (C) indicate significant differences from KitaakeX plants using Dunnett’s test (*P* < 0.05). Kitaake and KitaakeX were included as the susceptible and the resistant control, respectively.

To validate whether or not the *OsCSLD4* gene is responsible for the mutant phenotypes observed in FN2572, we set out to complement mutant FN2572 with the wild type *OsCSLD4* gene. We cloned and sequenced the genomic DNA of *OsCSLD4* from the Kitaake genome, including its native promoter. This gene was introduced into mutant FN2572 by *Agrobacterium*-mediated transformation. We obtained five independent T0 lines carrying the wild type *OsCSLD4* gene (*OsCSLD4*/FN2572) (Fig. 2B). When inoculated with *Xoo*, the five complemented lines showed resistance to *Xoo*, displaying lesion lengths similar to those of KitaakeX plants (Fig. 2B). The restoration of resistance to *Xoo* cosegregates with the *OsCSLD4* transgene in the T1 progeny of two transgenic complementation lines (Fig. 2C, Fig. S3). Only transgenic lines carrying the *OsCSLD4* gene (*OsCSLD4*/FN2572) conferred robust resistance to *Xoo* (Fig. 2C). The short stature of the mutant was also restored to wild type phenotype (Fig. 2A). These results validate our hypothesis that *OsCSLD4* is the responsible gene mutated in *sxi6* for the compromised *Xa21*-mediated immunity.

### Rice mutant FN2572 and W481 lines retain the ROS burst, MAPK activation, and marker gene *KO5* induction upon RaxX21-sY treatment similar to wild type

**Figure 3:**
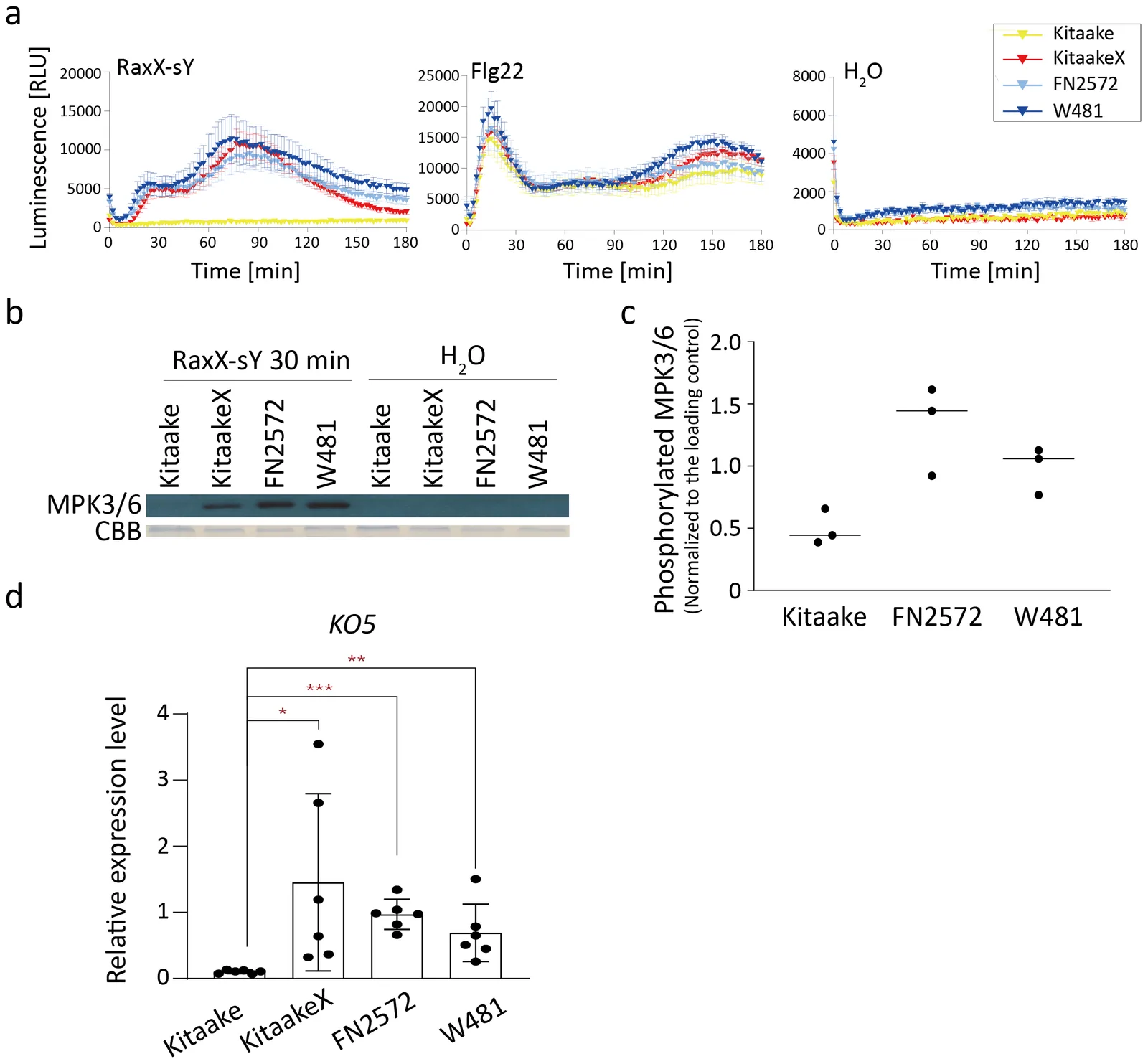
Hallmarks of the XA21-mediated immune responses are not affected in the *OsCSLD4* mutant. (A) Reactive oxygen species (ROS) accumulation (180 min) after peptide treatments. ROS production in leaves of Kitaake, KitaakeX and two rice mutants FN2572 and W481 treated with 500 nM RaxX-sY, 500 nM flg22, or H_2_O. RLU, relative light units. Curves represent mean ± SEM (*n* = 6). (B) Detection of the XA21 protein and the MAPK activity in RaxX-treated leaf tissue. The leave tissues from five-week-old plants were treated with 500 nM RaxX-sY or H_2_O for 30 min. Protein extracts were probed using an anti-p42/44 MAPK antibody in western blot analysis. Coomassie Brilliant Blue (CBB) staining of the membrane was included as a loading reference. (C) Quantitation of the phosphorylated MAK3/6 normalized to the loading control (CBB band) using ImageJ. (D) Induction of defense marker gene *KO5* expression in FN2572 and W481 mutants, and KitaakeX. Leaves from five-week-old plants were treated with 500 nM RaxX-sY for 9 h. Each bar represents mean ± SD of three independent biological replicates, with two technical replicates each (*n* = 6). Red asterisks indicate significant differences compared with wild-type Kitaake plants using Student’s *t*-test (*, *P* < 0.05; **, *P* < 0.01; ***, *P* < 0.001).

Sulfated RaxX21-sY peptide, a chemically synthesized tyrosine-sulfated 21-amino acid derivative of RaxX, the ligand for XA21, is able to activate XA21-mediated defense responses, including ROS burst, MAPK activation and defense-related marker gene expression in leaf clippings of KitaakeX plants (Pruitt et al., 2015). To assess whether *Oscsld4* mutations affect immune signaling, we monitored these defense responses upon RaxX21-sY treatment in mutants FN2572 and W481. As shown in Fig. 3A, when leaf clippings of mutants FN2572 and W481 were treated with 500 nM RaxX21-sY peptide, we observed a ROS burst in leaf clippings of mutants FN2572 and W481 at amplitudes similar to that of KitaakeX leaf clippings. Kitaake leaf clippings did not show a ROS burst upon RaxX21-sY treatment, nor did mock treatment with water (Fig. 3A). In addition, we found no significant differences in amplitudes of ROS bursts among leaf clippings of Kitaake, KitaakeX, and mutants FN2572 and W481 in responses to treatment with flagellin peptide flg22, a peptide known to induce immune responses in diverse plant species (Gómez-Gómez, Felix & Boller, 1999) (Fig. 3A). These results indicate that the *Oscsld4* mutations do not affect the ROS burst in plants treated with two distinct immunogens.

MAPK activation plays an important role in signal transduction under different stresses and leads to massive gene expression reprograming. We therefore assessed whether FN2572 and W481 are affected in MAPK activation in response to treatment with the RaxX21-sY peptide. We detected phosphorylated MAP kinases in proteins extracted from KitaakeX leaf clippings 30 min after treatment with RaxX21-sY as detected on Western blots using a phospho-p44/42 MAPK antibody. Our results reveal that phosphorylated MAPK proteins can be detected as soon as 30 min after RaxX21-sY treatment in KitaakeX and mutant FN2572 and W481 plants, but not in the Kitaake control (Fig. 3B). These results indicate that RaxX21-sY activates MAPK phosphorylation in an XA21-specific manner. Moreover, FN2572 and W481 displayed higher MAPK levels compared with the KitaakeX parental line (Fig. 3C). Similar results were observed in two replicate experiments.

We also monitored *PR* gene induction in mutants FN2572 and W481 by examining marker gene *KO5* expression upon RaxX21-sYpeptide treatment through reverse transcription quantitative PCR (RT-qPCR) in the leaf clipping assay as reported previously (Pruitt et al., 2015). Leaf clippings were collected 9 h post induction (hpi) with 500 nM RaxX21-sY. As shown in Fig. 3D, *KO5* was induced similarly in KitaakeX, FN2572 and W481 leaves, and accumulated to significantly higher levels in these three plants compared with the Kitaake control (*P*<0.05). These results demonstrate that *Oscsld4* mutations do not significantly reduce typical immune responses including ROS burst, MAPK activation and *KO5* induction, and suggest that ROS burst, MAPK activation and *KO5* induction are not sufficient to confer XA21-mediated immunity to *Xoo*.

### *OsCSLD4* affects immunity mediated by XA26

**Figure 4:**
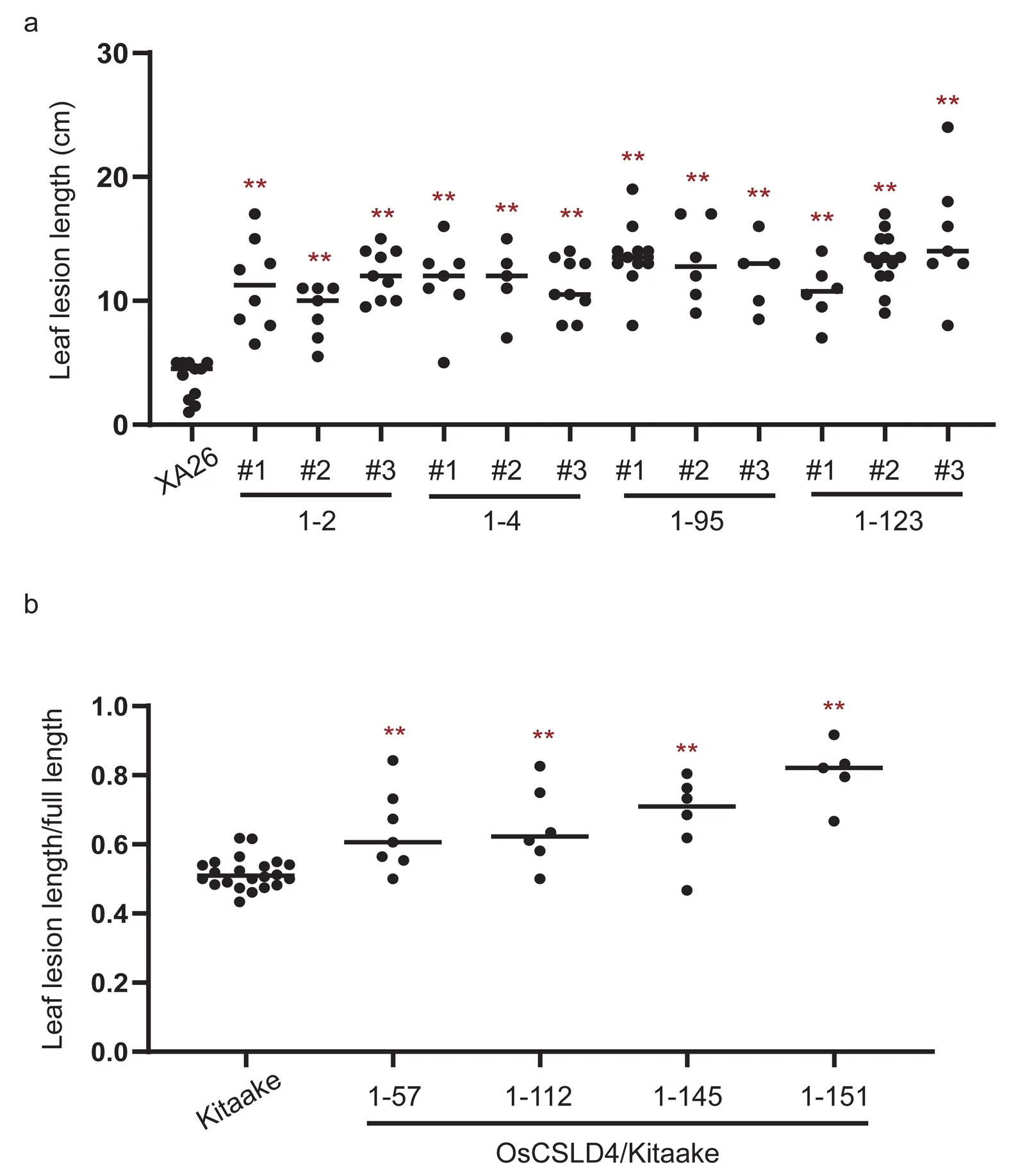
*OsCSLD4* is required for defense mediated by XA26 and the basal defense in rice plants. (A) Lesion lengths of *Xoo* strain PXO79-inoculated leaves from the F3 progeny of these four F2 homozygous *OsCSLD4* mutant lines (1–2, 1–4, 1–95, and 1–123) that contain XA26, but not XA21. Three biological replicates from the F3 progeny (*n* = 7–13). Red asterisks indicate significant differences from XA26 plants using Dunnett’s test (*P* < 0.05). (B) Lesion lengths of *Xoo*-inoculated leaves from these four F2 lines (1–57, 1–112, 1–145, and 1–151) were confirmed by genotyping to be homozygous in *OsCSLD4* mutation but lack both XA21 and XA26. Red asterisks indicate significant differences from Kitaake plants using Dunnett’s test (*P* < 0.01). Data points represent lesion lengths for 5–22 leaves.

To investigate whether *OsCSLD4* contributes to a broader range of immune responses, we first tested whether it is required for immunity mediated by XA26, a rice immune receptor conferring resistance to *Xoo* strain PXO79. We performed a cross between mutant FN2572 and a transgenic XA26 line (Liu et al., 2020), both in the Kitaake genetic background. Our goal was to generate lines that contain *XA26* and a homozygous *OsCSLD4* mutation, but that lacked the *Ubi-Xa21* gene. We obtained 2 independent F1 lines and self-pollinated both lines to produce F2 seeds for progeny analysis. After genotyping 119 lines of F2 progeny, we found 9 homozygous *Oscsld4* mutant F2 lines that contain *XA26*, but not *Xa21* (Figure. S4, S5, S6a, S6b). We chose 4 (line numbers,1-2, 1-4, 1-95, and1-123) of these F2 homozygous mutant lines for seed harvest and analysis. When the F3 progeny of these four lines were inoculated with *Xoo* strain PXO79, we found that *XA26* plants carrying the *OsCSLD4* mutation were significantly more susceptible to PXO79, displaying 2-4 fold longer lesions, compared with the XA26 control (Fig. 4A). This result suggests that *OsCSLD4* is also required for XA26-mediated immunity.

### *OsCSLD4* affects basal resistance in rice plants lacking major resistance genes

To assess whether *OsCSLD4* contributes to *Xoo* resistance in Kitaake lines lacking characterized immune receptors, we identified F2 progeny derived from the *OsCSLD4/XA21* and *XA26* cross that are homozygous for the *OsCSLD4* mutation but lack both *XA21* and *XA26*. We obtained four (1–57, 1–112, 1–145, and 1–151) such progeny lines (Figs. S4, S5, S6C, S6D). We inoculated these four lines and Kitaake control with *Xoo* and measured lesion lengths two weeks post inoculation. Because Kitaake leaves are fully susceptible to *Xoo* developing long lesions (around 15 cm long) and *OsCSLD4*/Kitaake leaves are relatively shorter than wild type Kitaake leaves. We measured both lesion length and leaf length, and divided the lesion length by the leaf length to calculate the portion that is diseased to represent the disease state. We found that these four *OsCSLD4*/Kitaake plants were all significantly more susceptible to *Xoo* compared with Kitaake control (Fig. 4B). This result suggests that the *OsCSLD4* mutation also affects basal resistance to *Xoo* in a rice variety that lacks two characterized resistance genes, *Xa21* and *Xa26*.

## Discussion

Cellulose is the dominant morphogenic polysaccharide in plant cell walls (Lampugnani et al., 2019; Cosgrove, 2024). In vascular plants, the highly conserved *CSLD* subfamily genes encode putative polysaccharides synthases that show sequence homology to the cellulose synthase CESA family (H). Loss-of-function mutations affecting members of the *CSLD* subfamily often lead to defects in plant development. For example, the *Arabidopsis* knockout mutant *csld5* has retarded stem and root growth (Bernal et al., 2007). Similarly, the *Arabidopsis csld2* and *csld3* mutants fail to develop root hairs (Favery et al., 2001; Wang et al., 2001; Bernal et al., 2008). These developmental defects are further enhanced by knocking out *AtCSLD5* under the *csld2* or *csld3* single mutant background, suggesting that *AtCSLD5* has overlapping functions with *AtCSLD2* and *AtCSLD3* (Yin et al., 2011). The rice ortholog of *AtCSLD5 is OsCSLD4* (Li et al., 2009). Loss-of-function mutations in *OsCSLD4* in rice resulted in similar developmental defects, including dwarfism and rolled leaves with narrow shape (Li et al., 2009; Hu et al., 2010; Yoshikawa et al., 2013; Ding et al., 2015; Shi et al., 2016), and a reduction of cellulose content in culms (Li et al., 2009). These studies demonstrate that the *CSLD* subfamily plays an essential role in plant development.

Consistent with previous reports, we observed developmental defects in the two rice mutants carrying predicted loss-of-function mutations in *OsCSLD4* (Fig. 2A). More importantly, we found that these mutants impair resistance to the bacterial pathogen *Xoo*, indicating a role for OsCSLD4 in plant immunity. More specifically, *OsCSLD4* is not only required for immunity to *Xoo* mediated by immune receptors XA21 and XA26, but also involved in basal resistance in Kitaake that does not contain any major resistance genes to *Xoo* (Figs. 1 and 4). These results suggest that *OsCSLD4* broadly contributes to the plant immune response. To test whether loss of *OsCSLD4* in rice affects defense signaling, we examined three typical defense hallmarks in the *OsCSLD4* knockout rice mutant upon treatment with RaxX-21sY, the cognate ligand of the immune receptor XA21. The ROS burst and induction of *KO5* remained unaffected in the mutants compared to the KitaakeX control, whereas phosphorylation of MAPK was enhanced. Our findings suggest that the susceptibility of these mutants to *Xoo* is intrinsically linked to these developmental and structural impairments rather than a specific defect in *R*-gene signaling. The fact that *oscsld4* mutants retain-and in the case of MAPK, even exhibit enhanced-immune responses (Fig. 3) indicates that the “sensing” and “signaling” apparatus of the plant is fully operational. However, the plant is unable to translate these signals into effective resistance. This strongly suggests that the developmental state of the *oscsld4* mutant creates a “permissive environment” for the pathogen. A cell wall with reduced cellulose content (as expected in a *csld* mutant) may be more easily degraded by *Xoo* secreted enzymes or offer less mechanical resistance to bacterial spread within the xylem and intercellular spaces. Therefore, we propose that OsCSLD4 does not play a direct regulatory role in XA21-mediated defense pathways. Instead, it is a prerequisite for a robust cellular “battlefield”. Without the structural integrity provided by normal OsCSLD4 function, even a fully activated immune system cannot effectively contain the pathogen colonization.

In a related study in barley, decreased cellulose content by knocking down the CSLD family member *HvCSLD2* is associated with increased susceptibility to the fungal pathogen powdery mildew due to enhanced hyphae penetration (Douchkov et al., 2016). This suggests that the structural defense provided by *CSLD* genes is likely broad-spectrum and not limited to bacterial pathogens. While the immune receptors XA21 and XA26 are specifically evolved to recognize *Xoo*, the cell wall integrity maintained by OsCSLD4 serves as a foundational barrier. The extreme susceptibility of *oscsld4* mutants observed in our study likely reflects a collapse of this general physical defense, which would theoretically render the plant more vulnerable to a wide array of pathogens beyond *Xoo*. Future studies testing *oscsld4* against fungal pathogens such as *Magnaporthe oryzae* would further define the breadth of this structural requirement. In addition, mutation of the cellulose synthase-like gene *CSLA9* in *Arabidopsis* inhibited *Agrobacterium*-mediated plant root transformation (Nam et al., 1999; Zhu et al., 2003).

While our results point to a structural basis for the increased susceptibility of *oscsld4* mutants, the precise cellular dynamics of this failure remain to be fully elucidated. Previous studies have confirmed that OsCSLD4 deficiency significantly reduces cellulose content and alters cell wall composition (Li et al., 2009). Future research employing transmission electron microscopy (TEM) and quantitative cell wall analysis will be essential to determine if specific layers of the xylem cell wall are thinner or more porous in these mutants. Furthermore, investigating whether the mutants fail to properly deposit callose or lignin at the infection site-despite having functional early immune signaling-would help clarify if OsCSLD4 is required for the ‘execution’ phase of structural defense. Detailed histological and microscopic tracking of *Xoo* progression within the mutant’s vascular system will also reveal whether the pathogen spreads more rapidly through compromised pit membranes or cell-to-cell junctions. Such studies will provide a more comprehensive understanding of how cell wall integrity acts as a frontline defense against vascular pathogens.

**Figure 5:**
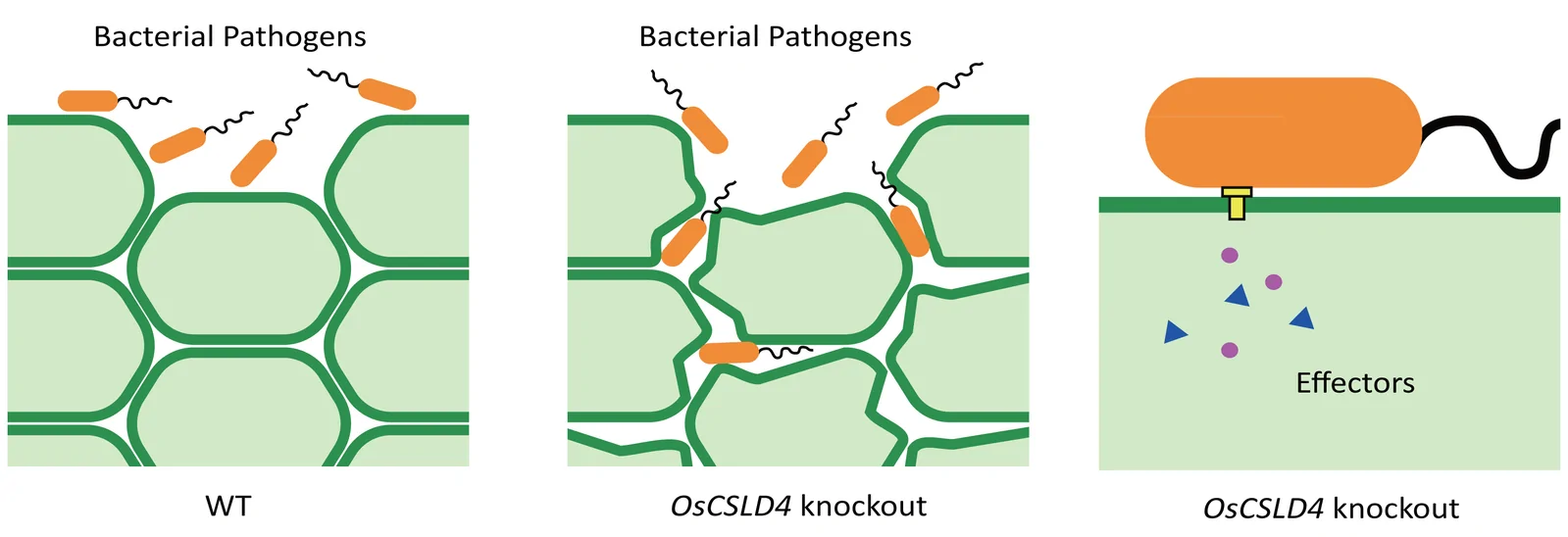
Model of *OsCSLD4* function in rice defense. A looser wall structure in *OsCSLD4* knockout rice may facilitate the invasion of pathogens and thereby enhance their ability to gain access to additional resources within the host. An altered cell wall structure in *OsCSLD4* knockout rice may make the cells more accessible to defense-suppressing effectors secreted by the pathogens. The model figure was created from scratch using Adobe Illustrator 2019.

In this study, we utilized the scissor-clipping inoculation method, which is a well-established and reproducible technique for evaluating rice resistance to the vascular pathogen *Xoo*. While this method bypasses early surface barriers such as hydathodes and stomata, it provides a direct assessment of the pathogen’s ability to multiply and spread systemically within the xylem vessels. The dramatic increase in lesion length and bacterial titers observed in *oscsld4* mutants—even when the initial entry barriers were bypassed—indicates that the structural integrity of the internal cell walls (particularly within or surrounding the vascular tissues) is compromised. We hypothesize that the change in wall structure may lead to increased susceptibility in the hosts by affecting two aspects of the pathogenicity (Fig. 5). First, a looser wall structure may facilitate the invasion of pathogens and thereby enhance their ability to gain access to additional resources within the host. This is the case for multiple lignin biosynthesis related genes (Xie et al., 2018). For example, phenylalanine ammonia-lyase (*PAL*) genes are important for lignin biosynthesis and *Xoo* and rice blast resistance (Tonnessen et al., 2015; Zhou et al., 2018). Second, an altered cell wall structure may make the cells more accessible to defense-suppressing effectors secreted by the pathogens. Such effectors promote virulence, a common strategy for most plant pathogens (Jones & Dangl, 2006). These hypotheses may be addressed in the future by investigating host defense responses to various pathogens in mutants with altered wall structures, and by tracking the invasion process. It is also possible that the *OsCSLD4* mutation weakens the host immune response by affecting components that remain to be identified.

A key question is whether the susceptibility of *oscsld4* is a specific defect in defense or a byproduct of general physiological weakness due to its severe developmental phenotypes, such as dwarfism and narrow leaves. Our data shows that while early immune signaling (ROS burst and MAPK activation) remains intact, the plant fails to restrict the pathogen. This suggests that the ‘physiological weakness’ in this mutant is specifically a ‘structural weakness’ of the cell wall. Given that *Xoo* is a vascular pathogen, any alteration in the cellulose matrix of the xylem vessels-even if primarily a developmental defect-will inherently reduce the physical barriers that normally limit bacterial movement. Therefore, the susceptibility of *oscsld4* likely stems from a loss of structural competence rather than a failure in pathogen recognition or initial signaling. The fact that the parent line KitaakeX remains resistant despite the wounding from the inoculation process further supports that the defect in oscsld4 is not just about ‘being unhealthy,’ but specifically about the inability of its compromised cell walls to function as an effective barrier.

## Conclusions

In summary, our characterization of the *OsCSLD4* gene reveals that cellulose-mediated cell wall integrity is a foundational component of rice defense against *Xoo*. While the loss of *OsCSLD4* compromises the efficacy of *XA21*- and *XA26*-mediated resistance, this effect is primarily due to the weakening of the physical barrier rather than the disruption of immune signaling. Together, these results suggest that the structural integrity maintained by OsCSLD4 is essential for the physical restriction of pathogen colonization. While OsCSLD4 is not required for the activation of early immune signaling, its role in cell wall biosynthesis provides the necessary structural framework to render those immune responses effective in containing the pathogen.

## Supplemental Information

10.7717/peerj.21562/supp-1 Supplemental Information 1 Raw Data.

10.7717/peerj.21562/supp-2 Supplemental Information 2 A 30 kb deletion on chromosome 12 co-segregates with the mutant W481 phenotype in an F3 population Five-week-old plants were inoculated with *Xoo*. Disease lesions were measured at 14 days after inoculation. Individual progeny were genotyped using a pair of primers targeting the *OsCSLD4* gene inside the 30 kb deletion, and the PCR results are shown below the bar graph.

10.7717/peerj.21562/supp-3 Supplemental Information 3 Sequence comparison of mutants W481 and FN2572 with KitaakeX parent Wild type parent KitaakeX and mutants W481 and FN2572 were whole-genome resequenced and their reads aligned to the Nippornbare reference genome. (A) An IGV snapshot of the 30 kb deletion in mutant W481. (B) Sequences of FN2572 progeny lines with phenotypes were aligned with wild type parent.

10.7717/peerj.21562/supp-4 Supplemental Information 4 Resistance to *Xoo.* cosegregates with the *OsCSLD4* transgene in T1 progeny Leaf lesion length data for 9 (#1 to #9) T1 plants derived from *OsCSLD4*/FN2572-8. Bars represent mean ± SD of lesion lengths measured 14 days post-inoculation (dpi). Red asterisks indicate significant differences from KitaakeX plants using Dunnett’s test.

10.7717/peerj.21562/supp-5 Supplemental Information 5 Genotyping of F1 progeny by using primers Ubi-3 and XA21R to test whether individual F1 progeny contain the *XA21* gene Progeny are derived from a cross between FN2572 (♀) and a transgenic XA26 line (♂), both in the Kitaake genetic background. Primer sequences are listed in Table S1.

10.7717/peerj.21562/supp-6 Supplemental Information 6 Genotyping of F1 progeny by using primers Ubi-3 and XA26R to test whether individual F1 progeny contain the *XA26* gene Progeny are derived from a cross between FN2572 (♀) and a transgenic XA26 line (♂). Primer sequences are listed in Table S1.

10.7717/peerj.21562/supp-7 Supplemental Information 7 Genotyping of F1 progeny by using two pairs of primers to test whether individual F1 progeny contain homozygous *OsCSLD4* mutation (A) PCR with primers 36890-Mutant-F/36890-Mutant-R that amplify mutant *Oscsld4*. (B) PCR with primers 36890-WT-F /36890-WT-R that amplify wild type *OsCSLD4* sequence. Progeny are derived from a cross between FN2572 (♀) and a transgenic XA26 line (♂). Homozygous mutant progeny that contain *XA26* but not *XA21* are highlighted with red check marks. Primer sequences are listed in Table S1.

10.7717/peerj.21562/supp-8 Supplemental Information 8 List of primers used in this study.

10.7717/peerj.21562/supp-9 Supplemental Information 9 MIQE Checklist.

## Additional Information and Declarations

### Data Availability

The following information was supplied regarding data availability: The raw data are available in the Supplemental Files.
