# Supplementary material for: The rice cellulose synthase-like D4 gene (OsCSLD4) is required for resistance to Xanthomonas pv. oryzae
Source: PeerJ. 2026 Sep 9;14:e21562. doi: 10.7717/peerj.21562 (PMC13570608; doi:10.7717/peerj.21562)
Supplement: Supplementary file 1 [file peerj-14-21562-s002.pdf]

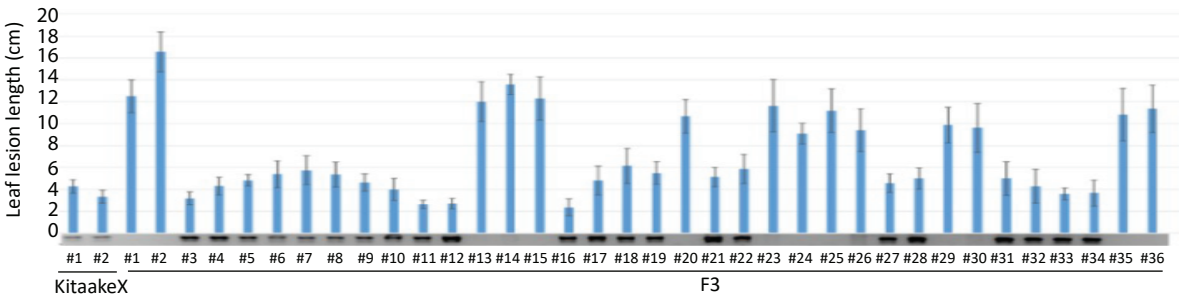

Leaf lesion length (cm)
20
18
16
14
12
10
8
6
4
2
0
#1 #2 #1 #2 #3 #4 #5 #6 #7 #8 #9 #10 #11 #12 #13 #14 #15 #16 #17 #18 #19 #20 #21 #22 #23 #24 #25 #26 #27 #28 #29 #30 #31 #32 #33 #34 #35 #36
KitaakeX
F3
