# Supplementary material for: The rice cellulose synthase-like D4 gene (OsCSLD4) is required for resistance to Xanthomonas pv. oryzae
Source: PeerJ. 2026 Sep 9;14:e21562. doi: 10.7717/peerj.21562 (PMC13570608; doi:10.7717/peerj.21562)
Supplement: Supplementary file 2 [file peerj-14-21562-s003.pdf]

a

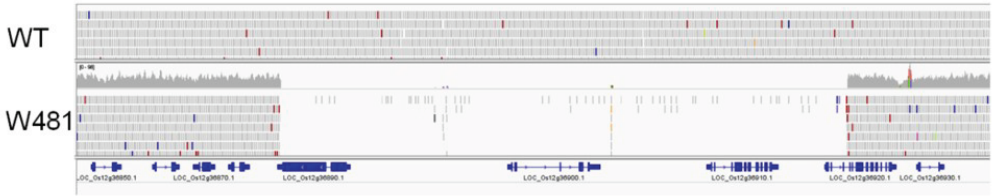

b

WT *Oscsld4* CGGTCGCTGGGGTCGACGCCCTCGAACCGCTGCGGGAACTGGACGAAGCACACGCGGTCG
FN2572-1 CGGTCG ------ GGTCGACGCCCTCGAACCGCTGCGGGAACTGGACGAAGCACACGCGGTCG
FN2572-2 CGGTCG ------ GGTCGACGCCCTCGAACCGCTGCGGGAACTGGACGAAGCACACGCGGTCG
FN2572-3 CGGTCG ------ GGTCGACGCCCTCGAACCGCTGCGGGAACTGGACGAAGCACACGCGGTCG
