# Supplementary material for: The rice cellulose synthase-like D4 gene (OsCSLD4) is required for resistance to Xanthomonas pv. oryzae
Source: PeerJ. 2026 Sep 9;14:e21562. doi: 10.7717/peerj.21562 (PMC13570608; doi:10.7717/peerj.21562)
Supplement: Supplementary file 3 [file peerj-14-21562-s004.pdf]

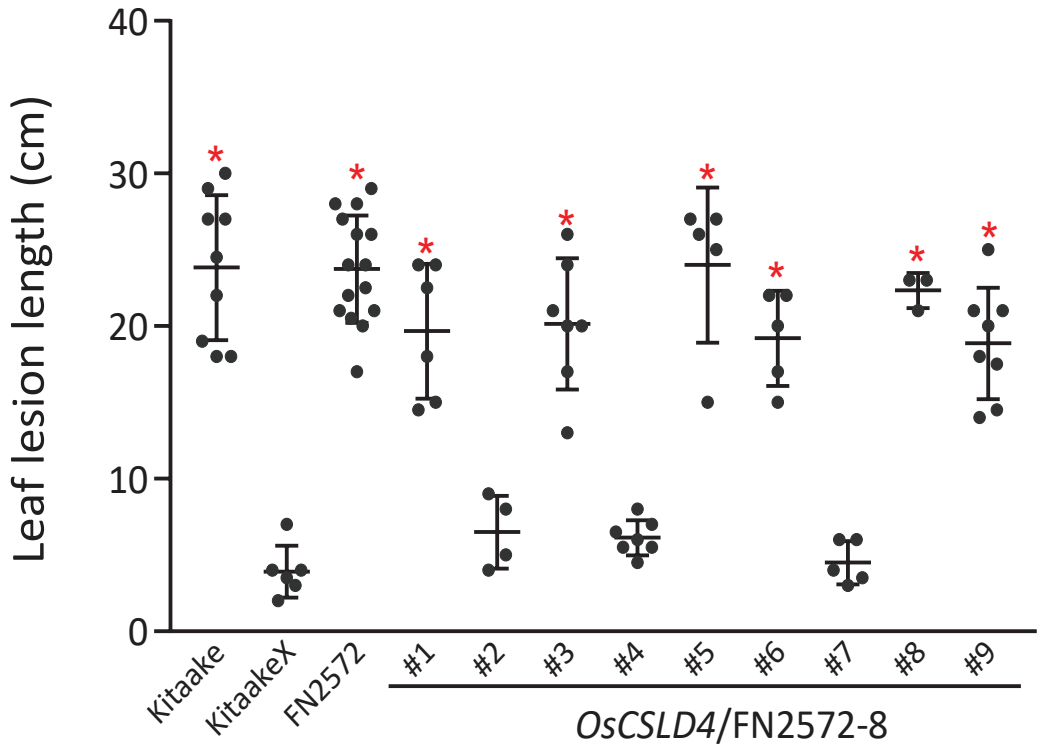
Leaf lesion length (cm)
40
30
20
10
0
Kitaake
KitaakeX
FN2572
#1
#2
#3
#4
#5
#6
#7
#8
#9
OsCSLD4/FN2572-8
