# Supplementary material for: The rice cellulose synthase-like D4 gene (OsCSLD4) is required for resistance to Xanthomonas pv. oryzae
Source: PeerJ. 2026 Sep 9;14:e21562. doi: 10.7717/peerj.21562 (PMC13570608; doi:10.7717/peerj.21562)
Supplement: Supplementary file 5 [file peerj-14-21562-s006.pdf]

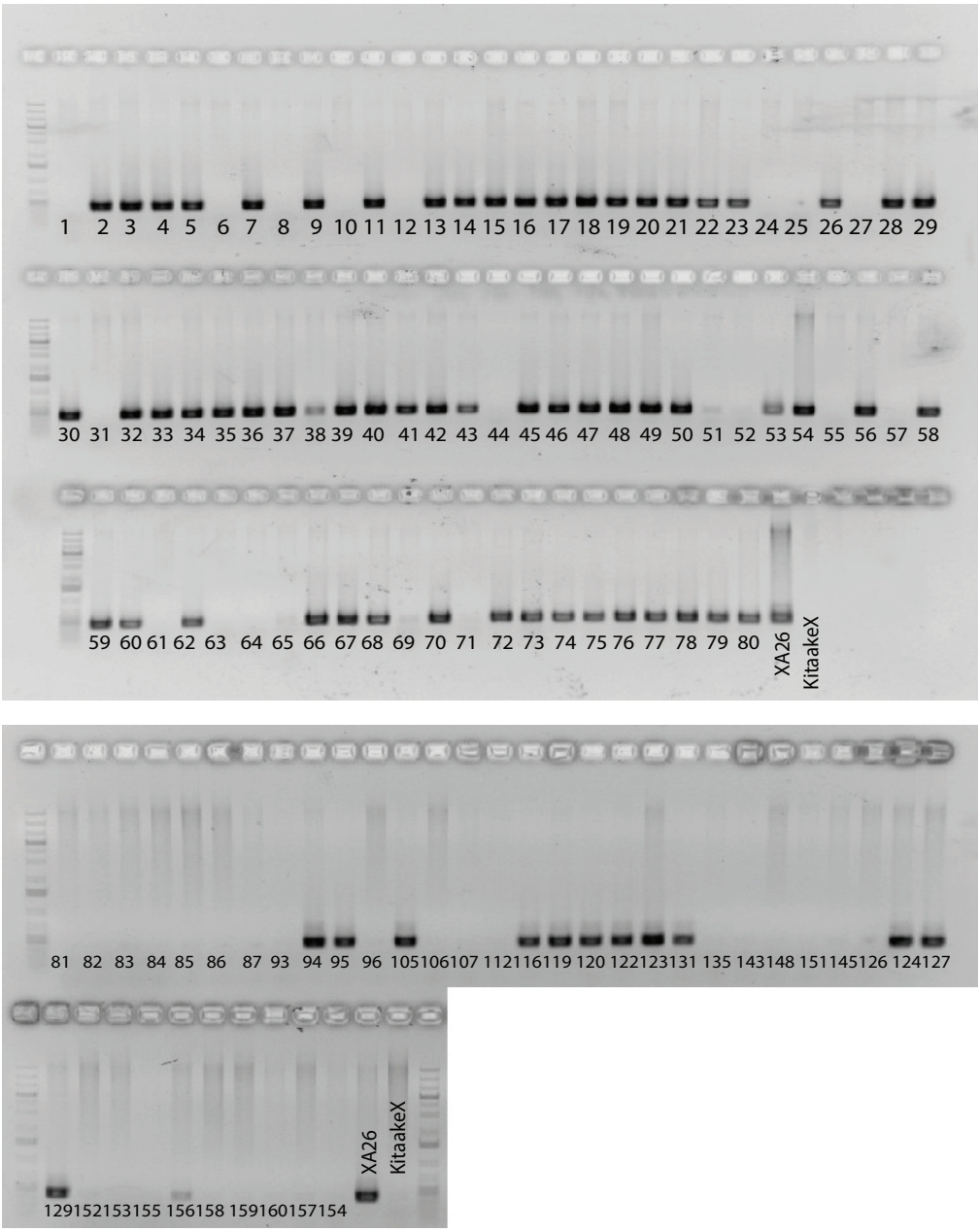
1 2 3 4 5 6 7 8 9 10 11 12 13 14 15 16 17 18 19 20 21 22 23 24 25 26 27 28 29
30 31 32 33 34 35 36 37 38 39 40 41 42 43 44 45 46 47 48 49 50 51 52 53 54 55 56 57 58
59 60 61 62 63 64 65 66 67 68 69 70 71 72 73 74 75 76 77 78 79 80
XA26
KitaakeX
81 82 83 84 85 86 87 93 94 95 96 105 106 107 112 116 119 120 122 123 131 135 143 148 151 145 126 124 127
129 152 153 155 156 158 159 160 157 154
XA26
KitaakeX
