# Supplementary material for: The rice cellulose synthase-like D4 gene (OsCSLD4) is required for resistance to Xanthomonas pv. oryzae
Source: PeerJ. 2026 Sep 9;14:e21562. doi: 10.7717/peerj.21562 (PMC13570608; doi:10.7717/peerj.21562)
Supplement: Supplementary file 7 [file peerj-14-21562-s008.doc]

**Table S1** List of primers used in this study.

| Primer name | Sequence |
| --- | --- |
| 12g36890F full length clone | CACCGCTACTCCTAGAGTATCCTCAC |
| 12g36890R full length clone | CTCCGTTTGTTTAGTTCACGCCA |
| 36890-Mutant-F | TTCGAGGGCGTCGACCCG |
| 36890-Mutant-R | CGTCCTCGGTGACGGAGCC |
| 36890-WT-F | CGTACAACGCCGGCGAGGA |
| 36890-WT-R | GTTGGCGTACCGGTCGCT |
| Ubi-3 | TGTCGATGCTCACCCTGTTGT |
| XA26-310R | AGCTGAGCTCTCCTTGGAGA |
| KO5F | GCTGGCTTCCAAACAAGAG |
| KO5R | GCCTCTTGATCAACGCGTTC |
